# Antiplatelet Therapy, Abdominal Aortic Aneurysm Progression, and Clinical Outcomes

**DOI:** 10.1001/jamanetworkopen.2023.47296

**Published:** 2023-12-12

**Authors:** Essa Hariri, Milad Matta, Habib Layoun, Osamah Badwan, Lorenzo Braghieri, A. Phillip Owens, Robert Burton, Rohan Bhandari, Doran Mix, John Bartholomew, David Schumick, Ayman Elbadawi, Samir Kapadia, Stanley L. Hazen, Lars G. Svensson, Scott J. Cameron

**Affiliations:** 1 Department of Internal Medicine, Cleveland Clinic Foundation, Cleveland, Ohio; 2 Division of Cardiology, Johns Hopkins Medicine, Baltimore, Maryland; 3 Cardiovascular Medicine, Section of Vascular Medicine, Heart, Vascular and Thoracic Institute, Cleveland Clinic Foundation, Cleveland, Ohio; 4 Division of Cardiovascular Medicine, Vanderbilt University Medical Center, Nashville, Tennessee; 5 Department of Cardiovascular Medicine, Heart, Vascular and Thoracic Institute, Cleveland Clinic Foundation, Cleveland, Ohio; 6 Department of Internal Medicine, Division of Cardiovascular Health and Disease, University of Cincinnati, Ohio; 7 Department of Surgery, Division of Vascular Surgery, University of Rochester Medical Center, New York; 8 Department of Cardiovascular and Metabolic Sciences, Lerner Research Institute of Case Western Reserve University, Cleveland, Ohio; 9 Division of Cardiology, Baylor College of Medicine, Houston, Texas; 10 Department of Cardiovascular Medicine, Section of Preventive Cardiology, Heart, Vascular and Thoracic Institute, Cleveland Clinic Foundation, Cleveland, Ohio; 11 Department of Hematology, Taussig Cancer Institute, Cleveland, Ohio

## Abstract

**Importance:**

Preclinical studies suggest a potential role for aspirin in slowing abdominal aortic aneurysm (AAA) progression and preventing rupture. Evidence on the clinical benefit of aspirin in AAA from human studies is lacking.

**Objective:**

To investigate the association of aspirin use with aneurysm progression and long-term clinical outcomes in patients with AAA.

**Design, Setting, and Participants:**

This was a retrospective, single-center cohort study. Adult patients with at least 2 available vascular ultrasounds at the Cleveland Clinic were included, and patients with history of aneurysm repair, dissection, or rupture were excluded. All patients were followed up for 10 years. Data were analyzed from May 2022 to July 2023.

**Main Outcomes and Measures:**

Clinical outcomes were time-to-first occurrence of all-cause mortality, major bleeding, or composite of dissection, rupture, and repair. Multivariable-adjusted Cox proportional-hazard regression was used to estimate hazard ratios (HR) for all-cause mortality, and subhazard ratios competing-risk regression using Fine and Gray proportional subhazards regression was used for major bleeding and composite outcome. Aneurysm progression was assessed by comparing the mean annualized change of aneurysm diameter using multivariable-adjusted linear regression and comparing the odds of having rapid progression (annual diameter change >0.5 cm per year) using logistic regression.

**Results:**

A total of 3435 patients (mean [SD] age 73.7 [9.0] years; 2672 male patients [77.5%]; 120 Asian, Hispanic, American Indian, or Pacific Islander patients [3.4%]; 255 Black patients [7.4%]; 3060 White patients [89.0%]; and median [IQR] follow-up, 4.9 [2.5-7.5] years) were included in the final analyses, of which 2150 (63%) were verified to be taking aspirin by prescription. Patients taking aspirin had a slower mean (SD) annualized change in aneurysm diameter (2.8 [3.0] vs 3.8 [4.2] mm per year; *P* = .001) and lower odds of having rapid aneurysm progression compared with patients not taking aspirin (adjusted odds ratio, 0.64; 95% CI, 0.49-0.89; *P* = .002). Aspirin use was not associated with risk of all-cause mortality (adjusted HR [aHR], 0.92; 95% CI, 0.79-1.07; *P* = .32), nor was aspirin use associated with major bleeding (aHR, 0.88; 95% CI, 0.76-1.03; *P* = .12), or composite outcome (aHR, 1.16; 95% CI, 0.93-1.45; *P* = .09) at 10 years.

**Conclusions:**

In this retrospective study of a clinical cohort of 3435 patients with objectively measured changes in aortic aneurysm growth, aspirin use was significantly associated with slower progression of AAA with a favorable safety profile.

## Key Points

**Question:**

Is aspirin use associated with progression and clinical outcomes of abdominal aortic aneurysm?

**Findings:**

In a cohort study of 3435 adults, aspirin use was associated with slower progression of abdominal aortic aneurysm, particularly in male participants and nonsmokers. Aspirin use was not associated with all-cause mortality, major bleeding, or composite of aneurysm dissection, rupture, or repair at 10 years.

**Meaning:**

These findings suggest that in patients with abdominal aortic aneurysm, the use of aspirin to slow aneurysm progression may be warranted.

## Teaser

This cohort study evaluates the association of acetylsalicylic acid use with aneurysm progression and long-term clinical outcomes in patients with abdominal aortic aneurysm.

## Introduction

Abdominal aortic aneurysm (AAA) is a common vascular disease linked to 1.3% of all deaths among men aged 65 to 85 years in developed countries.^1^ Despite its substantial risk of surgical mortality, guidelines currently recommend elective AAA repair if the diameter reaches 5.5 cm for symptomatic AAA or in cases of rapidly expanding AAA.^2,3^

Currently identified risk factors for AAA development and progression include age, male sex, hypertension, and smoking,^2^ with smoking being the most impactful modifiable risk factor.^4^ Risk factor control is currently the best available form of prevention for patients with AAA; thus there is an unmet need for medical therapies to slow the progression of AAA. Pharmacotherapy with β-blockers,^5^ angiotensin-converting enzyme inhibitors,^6^ doxycycline,^7^ and azithromycin^8^ have all failed to show benefit in limiting AAA progression or decreasing risk of rupture. One potential therapy that is associated with reduced AAA progression is metformin.^9,10,11^ Preclinical studies have shown that biomechanical platelet activation in a disturbed flow environment common to AAA is a pathophysiologic mechanism driving AAA development and growth, in addition to intramural thrombus formation.^12,13,14,15^ Although antiplatelet therapy was shown to reduce the intramural thrombi formation and aneurysm inflammatory response, and thus decrease the risk of rupture in animal models of AAA,^16^ the outcomes of aspirin in humans with AAA remains unclear.

Multiple studies have investigated the association of antiplatelet therapy with aneurysm progression in different vascular beds, including intracranial aneurysms^17^ and AAA, with conflicting evidence. Some studies found no association with AAA risk of rupture or growth rate,^18,19^ while others showed a decrease in AAA growth^20^ as well as the risk of rupture or dissection with aspirin use.^21^ Previous studies were, however, limited by small numbers of patients or aneurysms that are small in diameter. AAA has been considered a coronary artery disease equivalent in terms of cardiovascular events and mortality, and the use of aspirin at 75 mg to 162 mg daily in patients with AAA and intramural thrombus or penetrating ulcer has been given a class 2b (level of evidence: C) in the updated 2022 American College of Cardiology and American Heart Association guideline for the diagnosis and management of aortic disease.^22^ However, large clinical data on the role of aspirin in progression and outcomes of patients with AAA remain limited. Therefore, we sought to investigate the association of aspirin use with the progression of AAA and associated long-term clinical outcomes.

## Methods

### Study Design and Setting

**Figure 1.**
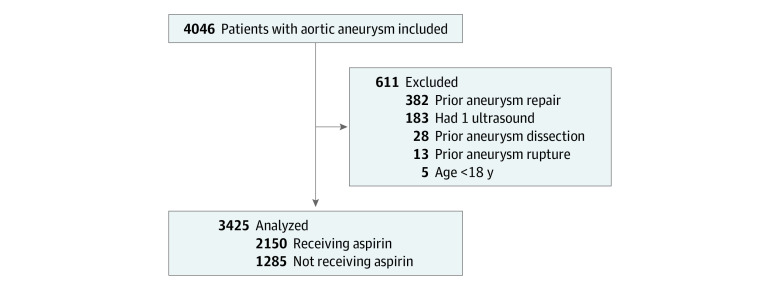
Flow Diagram for Study Population Selection

Data were collected on patients undergoing screening abdominal vascular ultrasound at the Cleveland Clinic vascular laboratory between 2010 and 2020. All patients selected had aortic aneurysm, defined as having a maximal aortic diameter in any dimension 3.0 cm or larger below the kidney arteries, irrespective of if the aneurysm was saccular or fusiform. We excluded all patients who were aged less than 18 years (5 patients), with a history of aneurysm endovascular or surgical repair (382 patients), dissection (28 patients), or rupture (13 patients). We also excluded patients who did not have 2 or more vascular ultrasounds (183 patients). Figure 1 summarizes the selection criteria for the population of patients included in the final analyses. The Cleveland Clinic institutional review board approved this study with a waiver of informed consent because data were deidentified. The study follows the Strengthening the Reporting of Observational Studies in Epidemiology (STROBE) reporting guideline for cohort studies and includes all the items recommended to be included in this manuscript.

### Study Populations

Patients were categorized into 2 groups according to use or nonuse of aspirin during their follow-up period. Aspirin use was defined as having at least 1 filled prescription for each participant during their follow-up duration. We collected prescription data using the medication reconciliation as documented in the electronic medical records, and we extracted data on over-the-counter use listed in the appointments for those taking them over-the-counter. Patient’s race was documented as self-reported race and extracted from their electronic medical records, and it was categorized as Black, White, and other (American Indian, Asian, Hispanic, or Pacific Islander). Race was assessed to provide a description of the racial distribution of the included population and adjust for this variable in all regression analyses.

### Data Collection

To ascertain our inclusion and exclusion criteria as well as the clinical outcomes of this study, we conducted an extensive manual medical record review of the following data for every patient: aspirin use and dosage, survival status, occurrence and time of aneurysm repair, aneurysm dissection, and aneurysm rupture. Measurements of abdominal aortic diameters were obtained from the vascular ultrasound laboratory database. Patient characteristics, including demographics, cardiovascular comorbidities and risk factors, connective tissue disorders, and smoking status, were extracted from the electronic medical records using *International Statistical Classification of Diseases and Related Health Problems, Tenth Revision (ICD-10)* codes (eTable 1 in Supplement 1). Medication use, including start and end dates for prescriptions, were directly extracted from the electronic medical records using Structured Query Language.

### AAA Diameter Measurements

Patients underwent AAA ultrasound scanning at the Cleveland Clinic vascular laboratory per the Cleveland Clinic Non-Invasive Vascular Laboratory Protocol with all scans performed by a registered vascular technician (RVT) and overread by a registered physician in vascular interpretation. Patients who had fasted were placed in the supine position, with the vascular technician starting at the level of the xiphoid process, in a transverse orientation, as a general survey of the aorta is done from its most proximal portion to the level of the iliac vessels. Any aneurysm, dissection, plaque, and/or other abnormality were noted. After the general survey was completed, the aorta was measured in the transverse plane at the proximal aorta (above celiac origin which is identified with color Doppler), at level of the kidney arteries, at mid aorta (midpoint between kidney arteries and distal measurement), and at distal aorta (within 2 cm of the aortic bifurcation), with the transducer kept perpendicular to the vessel to ensure accurate diameter measurements. Anteroposterior (AP) measurements were taken in longitudinal and transverse planes. The higher AP diameter was entered into the report. A width measurement was also taken in the transverse plane. All measurements were done outer wall to outer wall. Spectral Doppler waveforms were obtained at the proximal aorta, mid, and distal aorta at the level of the kidney and common iliac arteries and at the level of any stenosis. A 1.5- to 2.0-mm sample volume was used from the center stream of the vessel with an appropriate angle of less than 60 degrees.

### Study Outcomes

We investigated the association of aspirin use on the patients’ long-term clinical outcomes and aneurysm progression. All patients were followed up for 10 years. Clinical outcomes included time to first occurrence of all-cause mortality, major bleeding (types 2-5) according to Bleeding Academic Research Consortium criteria (eMethods in Supplement 1), and composite of dissection, rupture, and repair. Aneurysm progression was assessed by (1) odds of having rapid progression, defined as annual diameter change greater than 0.5 cm per year, and (2) the mean annualized change of aneurysm diameter, defined as the difference in the measured maximal diameters on the first and last abdominal vascular ultrasounds divided by the follow-up duration between the measurements.

### Statistical Analysis

All data were analyzed for equal variance (Brown-Forsythe) and normality (Shapiro-Wilk). Baseline characteristics were compared between study groups using a 2-sided *t* test (parametric) or Mann-Whitney *U* test (nonparametric) for continuous variables and analysis of variance for categorical variables. Continuous variables are represented as mean (SD) or median (IQR), and categorical variables are reported as proportions.

The associations of aspirin use with the clinical outcomes were assessed through survival analyses using the Kaplan-Meier nonparametric method. To account for differences in baseline characteristics and calculate survival estimates, we used multivariable-adjusted Cox proportional-hazard regression to estimate hazard ratios (HR) and competing-risk regression using Fine and Gray proportional subhazards model to estimate subhazard ratios for major bleeding and composite of aneurysm repair, dissection, or rupture, with mortality as competing event; both models were adjusted for demographics, comorbidities, smoking status, and initial measured aortic diameter. To evaluate for differences in aneurysm progression according to aspirin use, the median annualized change in maximal aortic diameter was compared between aspirin and nonaspirin users using multivariable-adjusted linear regression analyses. These analyses were further stratified by sex, baseline diameter, and smoking status given the association of both characteristics with aneurysm progression, and *P* value for interaction was calculated in these stratified and exploratory analyses. Regression diagnostics were performed to assess linear regression models assumptions. Additionally, we performed multivariable logistic regression to look at the association of aspirin use with rapid progression, defined as an increase in diameter of 0.5 cm or more per year.^22^

We finally performed sensitivity analyses using propensity matching to ascertain the association of aspirin use with both the clinical outcomes and aneurysm progression. Propensity matching was performed using the greedy matching strategy for aspirin use, and a propensity score for an aspirin user was considered matched to the closest propensity score of a nonaspirin user within a difference of 0.1. The method was repeated until all patients were matched or all propensity scores deviated by more than 0.1 between the groups. Propensity matching was assessed by determining covariate balance as measured by standardized mean difference in the selected variables between groups before and after propensity matching. Results of these comparisons demonstrated that adequate propensity matching was achieved between aspirin and nonaspirin users (eTable 2 and eFigure 1 in Supplement 1). Statistical significance was defined by *P* values less than .05. All analyses were conducted using Stata version 13.0 (Stata Corp) and R studio version 1.3.1073 (R Project for Statistical Computing). Data were analyzed from May 2022 to July 2023.

## Results

### Baseline Characteristics

A total of 3435 patients (mean [SD] age 73.7 [9.0] years) were included in the final analyses, and most patients were men (2672 participants [77.5%]). Overall, 120 patients (3.4%) were Asian, American Indian, Hispanic, or Pacific Islander; 255 patients (7.4%) were Black, and 3060 participants (89.0%) were White (Table 1). Patients were followed up for a median (IQR) of 4.9 (2.5-7.5) years. A total of 2150 (62.5%) were taking aspirin (1527 patients [71.0%] taking 81 mg aspirin ) with a median (IQR) duration of use of 10.6 (6.6-14.3) years. Only 18 patients had connective tissue disease, including Marfan syndrome (13 patients), Loes-Dietz syndrome (4 patients), and Ehlers-Danlos syndrome (1 patient). A total of 196 patients with syphilitic aortitis and 94 patients with Takayasu arteritis were also included in the analysis. There was no difference in the mean age, sex and race distribution, and smoking status according to aspirin use. However, patients receiving aspirin therapy had a smaller aneurysm diameter at baseline (3.51 vs 3.60 cm) and higher prevalence of comorbidities, including hypertension, hyperlipidemia, coronary artery disease, peripheral vascular disease, and chronic anemia. There was no difference in baseline prevalence of congestive heart failure, valvular heart diseases, chronic lung diseases, and diabetes. Moreover, patients taking aspirin were more likely to be taking cardiovascular medication such as platelet adenosine diphosphate P2Y12 receptor blockers, statins, metformin, and blood pressure medications. Additionally, there was no difference in the baseline characteristics of patients included in the final analyses (eTable 4 in Supplement 1) and those of the 118 excluded patients with 1 ultrasound.

**Table 1.** Baseline Characteristics of the Study Population

Characteristic	Participants, No. (%)	
	No aspirin (n = 1285)	Aspirin (n = 2150)
Age, mean (SD), y	73.7 (9.0)	73.7 (9.0)
Sex		
Female	280 (21.8)	483 (22.5)
Male	1005 (78.2)	1667 (77.5)
Race		
White	1143 (88.9)	1917 (89.2)
Black	97 (7.5)	158 (7.3)
Other^a^	45 (3.5)	75 (3.5)
Duration of aspirin use, median (IQR), y	NA	10.64 (6.62-14.29)
Initial aneurysm diameter, median (IQR), cm	3.60 (3.20-4.36)	3.51 (3.20-4.18)
Aspirin dose		
81 mg	NA	1527 (71.0)
162 mg	NA	0
324 mg	NA	457 (21.3)
Unknown	NA	166 (7.7)
Comorbidities		
Smoking	334 (26.0)	545 (25.3)
Hypertension	957 (74.5)	1671 (77.7)
Diabetes	309 (24.0)	499 (23.2)
Hyperlipidemia	997 (77.6)	1784 (83.0)
Coronary artery disease	278 (21.6)	1078 (50.1)
Peripheral vascular disease	331 (25.7)	833 (38.7)
Prior stroke	326 (25.4)	720 (33.5)
Congestive heart failure	185 (14.4)	301 (14.0)
Valvular heart disease	191 (14.9)	352 (16.4)
Chronic kidney disease	287 (22.3)	525 (24.4)
Chronic lung disease	70 (5.4)	116 (5.4)
Chronic anemia	589 (45.8)	1164 (54.1)
Nonatherosclerotic causes of AAA		
Marfan syndrome	8 (0.6)	5 (0.2)
Loes-Dietz syndrome	1 (0.1)	3 (0.1)
Ehlers-Danlos syndrome	0	1 (<0.01)
Syphilitic aorta	69 (5.4)	127 (5.9)
Takayasu arteritis	41 (3.2)	53 (2.5)
Medications		
P2Y12 receptor blocker	89 (6.9)	531 (24.7)
Anticoagulation	431 (33.5)	789 (36.7)
Statins	1055 (82.1)	2012 (93.6)
Metformin	215 (16.7)	462 (21.5)
ACE inhibitor/ARBs	898 (69.9)	1721 (80.0)
Calcium channel blockers	741 (57.7)	1389 (64.6)
β-Blockers	1015 (79.0)	1853 (86.2)

Abbreviations: AAA, abdominal aortic aneurysm; ACE, angiotensin-converting enzyme; ARB, angiotensin receptor blockers; NA, not applicable.

^a^ Other indicates patients with either American Indian, Asian, Hispanic, or Pacific Islander race.

### Association of Aspirin Use With Clinical Outcomes

**Figure 2.**
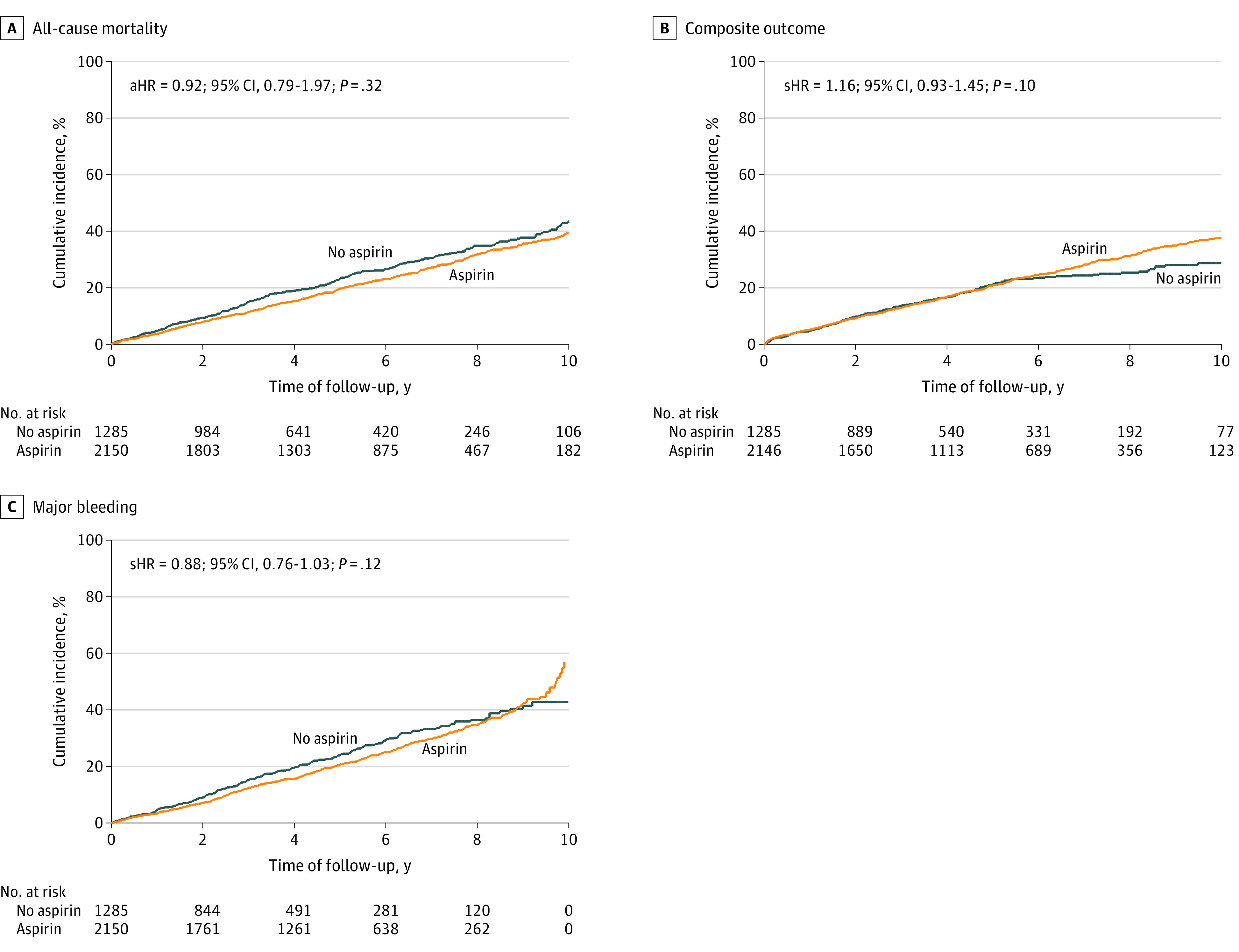
Kaplan-Meier Curves of the Cumulative Incidence of Clinical Outcomes Subhazard ratios (aHR) represent relative association of aspirin use with outcomes and calculated using competing risks regression with mortality as competing event. Subhazard ratios are adjusted for age, sex, smoking, comorbidities (hypertension, diabetes, chronic kidney disease, coronary artery disease, congestive heart failure, and anemia), medications (aspirin, statins, β-blockers, metformin, calcium channel blockers, angiotensin-converting enzyme inhibitors or angiotensin receptor blockers, anticoagulants, or P2Y12 receptor inhibitors), and baseline diameter.

Patients taking aspirin had 511 deaths (23.7%), 458 aneurysm repairs (21.3%), 11 aneurysm dissections (0.5%), and 8 aneurysm ruptures (0.4%), while patients not taking aspirin had 318 deaths (24.7%), 221 aneurysm repairs (17.1%), 4 aneurysm dissections (0.3%), and 5 aneurysm ruptures (0.5%). In a multivariable-adjusted Cox regression analysis, there was no significant difference in the incidence of all-cause mortality (adjusted HR [aHR], 0.92; 95% CI, 0.79-1.07; *P* = .32), major bleeding (aHR, 0.88; 95% CI, 0.76-1.03; *P* = .12), or composite of aneurysm repair, dissection, or rupture (adjusted subhazard ratio, 1.16; 95% CI, 0.93-1.45; *P* = .09) for patients taking aspirin, irrespective of sex or smoking status (Figure 2; eTable 2 in Supplement 1). In a sensitivity analysis that included a 1:1 propensity-matched group of 2170 patients, there was no significant difference in the incidence of clinical outcomes at 10 years according to aspirin use (eFigure 2 in Supplement 1).

### Association of Aspirin Use With AAA Progression

**Figure 3.**
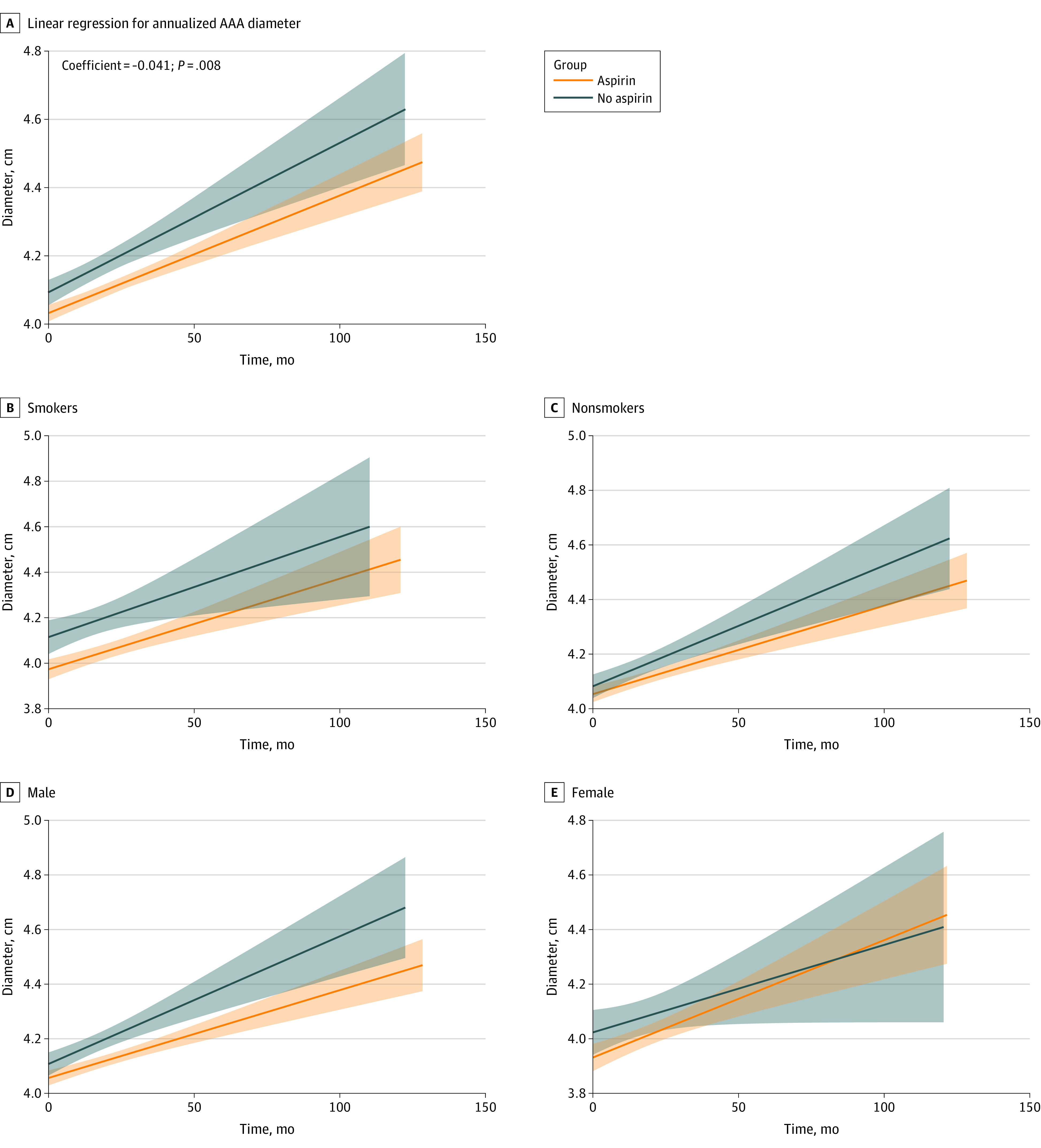
Change Over Time in Abdominal Aortic Aneurysm (AAA) Diameter According to Antiplatelet Therapy Patients taking long-term aspirin had significantly slower linear progression of AAA diameter over 10 years.

Patients taking aspirin also had a slower mean (SD) annualized change in aneurysm diameter compared with patients not taking aspirin (2.8 [3.0] vs 3.8 [4.2] mm per year; *P* = .001) (Figure 3; eTable 3 in Supplement 1). Similar findings were seen in sensitivity analysis using the 1:1 propensity-matched group patients, where those taking aspirin had a slower mean (SD) annualized change in aneurysm diameter compared with those not taking aspirin (2.8 [3.5] vs 3.5 [5.1] mm per year; *P* = .02). In a multivariable-adjusted linear regression model, aspirin use was negatively and significantly associated with the mean annualized change in the AAA diameter in the overall population (β = −0.041; 95% CI, −0.021 to 0.068; *P* = .001), and particularly in those with a baseline aortic diameter less than 5 cm (eTable 3 in Supplement 1). When stratifying according to sex and smoking status, aspirin use was associated with slower mean annualized change in aneurysm diameter compared with nonuse only among nonsmokers (β = −0.043; 95% CI, −0.018 to −0.071; *P* for interaction = 0.02) and males (β = −0.039; 95% CI, −0.022 to −0.066; *P* for interaction = 0.03). Stratified linear regression plots show similar results with a significant difference in AAA diameter progression where CI zones (gray zones around curves) do not intercept in nonsmokers and males (Figure 3). We compared the association of aspirin use with rapid (>5 mm per year) AAA progression (Table 2). Patients taking aspirin had 36% lower odds of having rapid aneurysm progression compared with patients not taking aspirin (adjusted odds ratio [aOR], 0.64; 95% CI, 0.49-0.89; *P* = .002), which was only seen among nonsmokers (OR, 0.63; 95% CI, 0.45-0.88; *P* = .008) and males (aOR, 0.64; 95% CI, 0.47-0.87; *P* = .005). We conducted sensitivity analysis by excluding patients with connective tissue disease and we found no difference in the association of aspirin use with clinical outcomes and AAA growth.

**Table 2.** Univariate and Multivariable Logistic Regression Analyses of Rapid Abdominal Aortic Aneurysm Progression According to Aspirin Use

Population	OR (95% CI)	
	Unadjusted	Multivariable adjusted^a^
Overall population	0.67 (0.50-0.86)	0.64 (0.49-0.89)
Smokers (n = 879)	0.63 (0.39-1.01)	0.66 (0.46-1.09)
Nonsmokers (n = 2556)	0.68 (0.49-0.93)	0.63 (0.45-0.88)
Male participants (n = 2672)	0.65 (0.49-0.88)	0.64 (0.47-0.87)
Female participants (n = 763)	0.67 (0.37-1.22)	0.74 (0.39-1.41)

Abbreviation: OR, odds ratio.

^a^ Adjusted for age, sex, smoking, comorbidities (hypertension, diabetes, chronic kidney disease, dialysis, coronary artery disease, congestive heart failure, and anemia), medications (aspirin, statins, β-blockers, calcium channel blockers, angiotensin-converting enzyme inhibitors/angiotensin receptor blockers, anticoagulants, and P2Y12 receptor inhibitors), and baseline diameter.

## Discussion

This is the largest contemporary cohort study to evaluate the role of aspirin in progression and long-term clinical outcomes of AAA. Aspirin use was associated with slower progression of AAA in nonsmokers and in males. However, there was no difference in the incidence of all-cause mortality, major bleeding and progression to aneurysm repair, aortic dissection, or rupture with aspirin use.

Unlike blood pressure control and statin use, which carry a class 1 recommendation because of their positive impact on probable concomitant coronary artery disease, aspirin use carries only a class 2b recommendation for AAA in the updated 2022 American College of Cardiology and American Heart Association Guidelines for the Diagnosis and Management of Aortic Disease.^22^ The guidelines also highlight the need for more clinical and mechanistic data to support using aspirin beyond its protective effect on probable coexisting coronary artery atherosclerosis. Very few studies reported the association of aspirin with clinical outcomes in patients with AAA, and most are limited by small sample sizes, heterogenous populations including thoracic and abdominal aneurysms or postrepair patients, limited follow-up, administrative databases, or use of *ICD*-coded diagnoses for their reporting on aspirin use and study outcomes.^23,24,25,26^ Our findings are similar to a large meta-analysis of studies from different countries showing no significant association of antiplatelet therapy with aneurysm growth or rupture but with notable heterogeneity in the included studies in terms of available measurement data for AAA and reporting on the use of antiplatelet medications.^27^ Interestingly, the event rate for aneurysm rupture was rare in all the included studies, similar to ours. In a small study by Bailey et al,^28^ conducted on 145 male patients, it was noted that patients with AAA treated with aspirin have a similar survival over 5 years compared with those not taking aspirin therapy.^28^ In contrast, among patients admitted for AAA rupture, preadmission aspirin use was found to be associated with higher very short-term (30-day) mortality among 4010 patients admitted with ruptured AAA from the Danish National Registry.^18,29^ Alongside our observation of similar long-term (10-year) survival in patients with AAA taking aspirin compared with those not taking aspirin, we demonstrate the relative safety of aspirin by showing no increased risk for major bleeding, aneurysm dissection, or rupture.

Our findings of slower AAA progression with aspirin use are concordant with preclinical and translational studies showing a role of platelet activation in growth and rupture of AAA.^14,30,31^ However, clinical studies reporting objective measures of AAA progression are limited. One study from Lindholt et al^20^ that included 148 patients with small AAA aneurysms (between 30-48 mm) who were followed up for a median of 6.6 years showed that the rate of expansion was lower in the aspirin users in patients with initial aortic aneurysm size between 40 to 49 mm. In the same study, there was no difference in the risk of undergoing surgical repair according to aspirin use, irrespective of initial aneurysm diameter. In addition, another well-designed study failed to show an association of the platelet P2Y12 receptor antagonist ticagrelor with growth of small AAA of around 3.5 cm.^19^ This study, however, had a shorter follow-up duration and raises the possibility that small AAA do not generate enough biomechanical stress on platelets to activate them in a way larger AAA would by disturbed flow and that the mechanism by which platelets are inhibited and treatment duration are of equal importance to the AAA size. Nonetheless, our previous work in animal models demonstrated that platelet-derived mediators accelerate AAA growth which are blocked by introducing aspirin once an aneurysm is detected,^14^ and our current data in a large clinical population evaluated over a decade provide clear evidence that aspirin use may reduce growth and progression of AAA in select patient populations.

Our further findings highlight the complex pathophysiological nature of AAA progression. According to our study, progression of AAA is modulated by sex and smoking status. It is well known that smoking is the strongest modifiable risk factor for AAA development, and the mechanism by which smoking modulates AAA progression could be associated with tunica media remodeling through epigenetic mechanisms and activation of proinflammatory cascades, including zinc endopeptidases in the matrix metalloproteinase family of enzymes,^32^ which aspirin may or may not alter.^33,34^ Sex differences in AAA development have also been investigated. Even though AAA is 4 to 6 times more likely to occur in male patients, female patients tend to have worse outcomes once AAA diagnosis is established as they experience a higher risk of aneurysm rupture and worse outcomes after repair compared with men, with rupture often occurring at a smaller diameter.^35^ It is therefore plausible that our findings suggest aspirin's role in AAA in women is limited due to the aggressive nature of the disease in this population.

### Strengths and Limitations

Our study has several strengths. The large sample size available and long-term follow-up in addition to manual medical record review allowed for limited measurement error and ability to perform multivariable adjustment and propensity matching to report associations of aspirin with long-term clinical outcomes in a unique population of native AAA. Furthermore, the availability of objective assessment of aneurysm diameters via ultrasound provided more accurate assessment on aneurysm growth than relying on *ICD-9* or *ICD-10* codes or other clinical indicators. All vascular ultrasounds at our institution are performed by RVTs, following the Cleveland Clinic vascular laboratory protocol, making image acquisition and interpretation standardized, thus limiting variability.

This study has limitations. Measurements via ultrasonography may be subject to intrinsic variability in obtained measurements by approximately 2 to 4 mm as previously reported.^36^ As with any retrospective observational study, we are limited by the inability to ascertain any causal associations as well as selection bias. Second, this was a single-center study with our study population, which could limit generalizability of our findings. Another potential limitation is the risk of misclassification of aspirin use and the possibility of over-the-counter aspirin use that was not documented in clinician notes and difficult to fully reconcile. The filling of an aspirin prescription was used as a proxy measure of aspirin use.

## Conclusions

In conclusion, we found that aspirin use was associated with slower progression of AAA with a favorable safety profile in a large retrospective single center. Given the myriad of preclinical and clinical data suggesting a role of platelet activation and inhibition in modulating this disease process, randomized clinical data are warranted to ascertain the role of aspirin in managing AAA.

## Notes

Supplement 1.
eMethods
**eTable 1.** *ICD-10 *Codes for Included Patient Variables **eTable 2. **Univariate and Multivariable-Adjusted Regression Analyses of All-Cause Mortality and Composite of Aneurysm Repair, Dissection, or Rupture, According to Aspirin Use **eTable 3. **Univariate and Multivariable Linear Regression Analyses for the Annualized Change Abdominal Aortic Aneurysm Diameter According to Antiplatelet Use **eTable 4. **Baseline Characteristics of the Excluded Population **eFigure 1. **Love Plot for Covariate Balance in the 1:1 Propensity-Matched Group of Aspirin and Non-Aspirin Users **eFigure 2. **Kaplan-Meier Curves of the Cumulative Incidence of Study Outcomes in a 1:1 Propensity-Matched Cohort

Supplement 2.
Data Sharing Statement

## References

[1] Sakalihasan N, Limet R, Defawe OD. Abdominal aortic aneurysm. Lancet. 2005;365(9470):1577-1589. doi:10.1016/S0140-6736(05)66459-8 15866312

[2] Wanhainen A, Verzini F, Van Herzeele I, ; Esvs Guidelines Committee. Editor’s choice—European Society for Vascular Surgery (ESVS) 2019 clinical practice guidelines on the management of abdominal aorto-iliac artery aneurysms. Eur J Vasc Endovasc Surg. 2019;57(1):8-93. doi:10.1016/j.ejvs.2018.09.020 30528142

[3] Landon BE, O’Malley AJ, Giles K, Cotterill P, Schermerhorn ML. Volume-outcome relationships and abdominal aortic aneurysm repair. Circulation. 2010;122(13):1290-1297. doi:10.1161/CIRCULATIONAHA.110.949172 20837892

[4] Nordon IM, Hinchliffe RJ, Loftus IM, Thompson MM. Pathophysiology and epidemiology of abdominal aortic aneurysms. Nat Rev Cardiol. 2011;8(2):92-102. doi:10.1038/nrcardio.2010.180 21079638

[5] Rughani G, Robertson L, Clarke M. Medical treatment for small abdominal aortic aneurysms. Cochrane Database Syst Rev. 2012;(9):CD009536. doi:10.1002/14651858.CD009536 22972146 PMC11707027

[6] Bicknell CD, Kiru G, Falaschetti E, Powell JT, Poulter NR; AARDVARK Collaborators. An evaluation of the effect of an angiotensin-converting enzyme inhibitor on the growth rate of small abdominal aortic aneurysms: a randomized placebo-controlled trial (AARDVARK). Eur Heart J. 2016;37(42):3213-3221. doi:10.1093/eurheartj/ehw257 27371719 PMC5181384

[7] Baxter BT, Matsumura J, Curci JA, ; N-TA3CT Investigators. Effect of doxycycline on aneurysm growth among patients with small infrarenal abdominal aortic aneurysms: a randomized clinical trial. JAMA. 2020;323(20):2029-2038. doi:10.1001/jama.2020.5230 32453369 PMC7251450

[8] Karlsson L, Gnarpe J, Bergqvist D, Lindbäck J, Pärsson H. The effect of azithromycin and Chlamydophilia pneumonia infection on expansion of small abdominal aortic aneurysms–a prospective randomized double-blind trial. J Vasc Surg. 2009;50(1):23-29. doi:10.1016/j.jvs.2008.12.048 19563951

[9] Turowicz A, Kobecki J, Laskowska A, Wojciechowski J, Świątkowski F, Chabowski M. Association of metformin and abdominal aortic aneurysm repair outcomes. Ann Vasc Surg. 2021;75:390-396. doi:10.1016/j.avsg.2021.02.048 33826959

[10] Raffort J, Hassen-Khodja R, Jean-Baptiste E, Lareyre F. Relationship between metformin and abdominal aortic aneurysm. J Vasc Surg. 2020;71(3):1056-1062. doi:10.1016/j.jvs.2019.08.270 31727461

[11] Yuan Z, Heng Z, Lu Y, Wei J, Cai Z. The protective effect of metformin on abdominal aortic aneurysm: a systematic review and meta-analysis. Front Endocrinol (Lausanne). 2021;12:721213. doi:10.3389/fendo.2021.721213 34394010 PMC8355809

[12] Hansen KB, Arzani A, Shadden SC. Mechanical platelet activation potential in abdominal aortic aneurysms. J Biomech Eng. 2015;137(4):041005. doi:10.1115/1.4029580 25588057 PMC4321116

[13] Milne AA, Adam DJ, Murphy WG, Ruckley CV. Effects of asymptomatic abdominal aortic aneurysm on the soluble coagulation system, platelet count and platelet activation. Eur J Vasc Endovasc Surg. 1999;17(5):434-437. doi:10.1053/ejvs.1998.0790 10329529

[14] Morrell CN, Mix D, Aggarwal A, . Platelet olfactory receptor activation limits platelet reactivity and growth of aortic aneurysms. J Clin Invest. 2022;132(9):e152373. doi:10.1172/JCI152373 35324479 PMC9057618

[15] Touat Z, Ollivier V, Dai J, . Renewal of mural thrombus releases plasma markers and is involved in aortic abdominal aneurysm evolution. Am J Pathol. 2006;168(3):1022-1030. doi:10.2353/ajpath.2006.050868 16507915 PMC1606522

[16] Liu O, Jia L, Liu X, . Clopidogrel, a platelet P2Y12 receptor inhibitor, reduces vascular inflammation and angiotensin II induced-abdominal aortic aneurysm progression. PLoS One. 2012;7(12):e51707. doi:10.1371/journal.pone.0051707 23284748 PMC3527447

[17] Zanaty M, Roa JA, Nakagawa D, . Aspirin associated with decreased rate of intracranial aneurysm growth. J Neurosurg. 2019;1-8. 31662579 10.3171/2019.6.JNS191273

[18] Wemmelund H, Jørgensen TM, Høgh A, Behr-Rasmussen C, Johnsen SP, Lindholt JS. Low-dose aspirin and rupture of abdominal aortic aneurysm. J Vasc Surg. 2017;65(3):616-625.e4. doi:10.1016/j.jvs.2016.04.061 27460909

[19] Wanhainen A, Mani K, Kullberg J, . The effect of ticagrelor on growth of small abdominal aortic aneurysms-a randomized controlled trial. Cardiovasc Res. 2020;116(2):450-456. 31135888 10.1093/cvr/cvz133

[20] Lindholt JS, Sorensen HT, Michel JB, Thomsen HF, Henneberg EW. Low-dose aspirin may prevent growth and later surgical repair of medium-sized abdominal aortic aneurysms. Vasc Endovascular Surg. 2008;42(4):329-334. doi:10.1177/1538574408315205 18728038

[21] Elbadawi A, Omer M, Ogunbayo G, . antiplatelet medications protect against aortic dissection and rupture in patients with abdominal aortic aneurysms. J Am Coll Cardiol. 2020;75(13):1609-1610. doi:10.1016/j.jacc.2020.02.012 32241378 PMC7138435

[22] Isselbacher EM, Preventza O, Hamilton Black J III, . 2022 ACC/AHA guideline for the diagnosis and management of aortic disease: a report of the American Heart Association/American College of Cardiology joint committee on clinical practice guidelines. Circulation. 2022;146(24):e334-e482. doi:10.1161/CIR.0000000000001106 36322642 PMC9876736

[23] Granath C, Freiholtz D, Bredin F, Olsson C, Franco-Cereceda A, Björck HM. Acetylsalicylic acid is associated with a lower prevalence of ascending aortic aneurysm and a decreased aortic expression of cyclooxygenase 2. J Am Heart Assoc. 2022;11(9):e024346. doi:10.1161/JAHA.121.024346 35470674 PMC9238591

[24] Chen CY, Huang JW, Tzu-Chi Lee C, Lai WT, Huang YB. Long-term outcome of patients with aortic aneurysms taking low-dose aspirin: a population-based cohort study. J Investig Med. 2013;61(6):1004-1012. doi:10.2310/JIM.0b013e318297d0f9 23703144

[25] Eldrup N, Budtz-Lilly J, Laustsen J, Bibby BM, Paaske WP. Long-term incidence of myocardial infarct, stroke, and mortality in patients operated on for abdominal aortic aneurysms. J Vasc Surg. 2012;55(2):311-317. doi:10.1016/j.jvs.2011.08.046 22051869

[26] Thompson A, Cooper JA, Fabricius M, Humphries SE, Ashton HA, Hafez H. An analysis of drug modulation of abdominal aortic aneurysm growth through 25 years of surveillance. J Vasc Surg. 2010;52(1):55-61.e2. doi:10.1016/j.jvs.2010.02.012 20620765

[27] Sweeting MJ, Thompson SG, Brown LC, Powell JT; RESCAN collaborators. Meta-analysis of individual patient data to examine factors affecting growth and rupture of small abdominal aortic aneurysms. Br J Surg. 2012;99(5):655-665. doi:10.1002/bjs.8707 22389113

[28] Bailey MA, Aggarwal R, Bridge KI, . Aspirin therapy is associated with less compact fibrin networks and enhanced fibrinolysis in patients with abdominal aortic aneurysm. J Thromb Haemost. 2015;13(5):795-801. doi:10.1111/jth.12872 25660763

[29] Wemmelund H. Abdominal aortic aneurysms pharmacoepidemiological studies. Dan Med J. 2017;64(5):B5375. 28552095

[30] Gąsecka A, Zawadka M, Burban A, . Pre-operative platelet reactivity is a strong, independent predictor of bleeding complications after branched endovascular thoracoabdominal aortic aneurysm repair. Platelets. 2022;33(4):577-585. doi:10.1080/09537104.2021.1961708 34355639

[31] Weng JC, Wang J, Li H, ; Small Unruptured Aneurysms Study Group. Aspirin and growth of small unruptured intracranial aneurysm: results of a prospective cohort study. Stroke. 2020;51(10):3045-3054. doi:10.1161/STROKEAHA.120.029967 32878566

[32] Norman PE, Curci JA. Understanding the effects of tobacco smoke on the pathogenesis of aortic aneurysm. Arterioscler Thromb Vasc Biol. 2013;33(7):1473-1477. doi:10.1161/ATVBAHA.112.300158 23685557 PMC3683352

[33] Longo GM, Xiong W, Greiner TC, Zhao Y, Fiotti N, Baxter BT. Matrix metalloproteinases 2 and 9 work in concert to produce aortic aneurysms. J Clin Invest. 2002;110(5):625-632. doi:10.1172/JCI0215334 12208863 PMC151106

[34] Shen M, Lee J, Basu R, . Divergent roles of matrix metalloproteinase 2 in pathogenesis of thoracic aortic aneurysm. Arterioscler Thromb Vasc Biol. 2015;35(4):888-898. doi:10.1161/ATVBAHA.114.305115 25657308

[35] Desai M, Choke E, Sayers RD, Nath M, Bown MJ. Sex-related trends in mortality after elective abdominal aortic aneurysm surgery between 2002 and 2013 at National Health Service hospitals in England: less benefit for women compared with men. Eur Heart J. 2016;37(46):3452-3460. doi:10.1093/eurheartj/ehw335 27520304

[36] Singh K, Bønaa KH, Solberg S, Sørlie DG, Bjørk L. Intra- and interobserver variability in ultrasound measurements of abdominal aortic diameter. The Tromsø Study. Eur J Vasc Endovasc Surg. 1998;15(6):497-504. doi:10.1016/S1078-5884(98)80109-3 9659884

