# Supplementary material for: Antiplatelet Therapy, Abdominal Aortic Aneurysm Progression, and Clinical Outcomes
Source: JAMA Netw Open. 2023 Dec 12;6(12):e2347296. doi: 10.1001/jamanetworkopen.2023.47296 (PMC10716735; doi:10.1001/jamanetworkopen.2023.47296)
Supplement: Supplement 1. — eMethods eTable 1. ICD-10 Codes for Included Patient Variables eTable 2. Univariate and Multivariable-Adjusted Regression Analyses of All-Cause Mortality and Composite of Aneurysm Repair, Dissection, or Rupture, According to Aspirin Use eTable 3. Univariate and Multivariable Linear Regression Analyses for the Annualized Change Abdominal Aortic Aneurysm Diameter According to Antiplatelet Use eTable 4. Baseline Characteristics of the Excluded Population eFigure 1. Love Plot for Covariate Balance in the 1:1 Propensity-Matched Group of Aspirin and Non-Aspirin Users eFigure 2. Kaplan-Meier Curves of the Cumulative Incidence of Study Outcomes in a 1:1 Propensity-Matched Cohort [file jamanetwopen-e2347296-s001.pdf]

Supplemental Online Content

Hariri E, Matta M, Layoun H, et al. Antiplatelet therapy, abdominal aortic aneurysm progression, and clinical outcomes. *JAMA Netw Open*. 2023;6(12):e2347296. doi: 10.1001/jamanetworkopen.2023.47296

eMethods
eTable 1. *ICD-10* Codes for Included Patient Variables
eTable 2. Univariate and Multivariable-Adjusted Regression Analyses of All-Cause Mortality and Composite of Aneurysm Repair, Dissection, or Rupture, According to Aspirin Use
eTable 3. Univariate and Multivariable Linear Regression Analyses for the Annualized Change Abdominal Aortic Aneurysm Diameter According to Antiplatelet Use
eTable 4. Baseline Characteristics of the Excluded Population
eFigure 1. Love Plot for Covariate Balance in the 1:1 Propensity-Matched Group of Aspirin and Non-Aspirin Users
eFigure 2. Kaplan-Meier Curves of the Cumulative Incidence of Study Outcomes in a 1:1 Propensity-Matched Cohort

This supplemental material has been provided by the authors to give readers additional information about their work.

© 2023 Hariri E et al. *JAMA Network Open.*

eMethods

BARC (Bleeding Academic Research Consortium) Definition

Type 1: bleeding that is not actionable and does not cause the patient to seek an unscheduled performance of studies, hospitalization, or treatment by a health care professional; it may include episodes leading to self-discontinuation of medical therapy by the patient without consulting a health care professional.

Type 2: any overt, actionable sign of hemorrhage (e.g., more bleeding than would be expected for a clinical circumstance, including bleeding found by imaging alone) that does not fit the criteria for type 3, type 4, or type 5 but does meet at least one of the following criteria: requiring nonsurgical, medical intervention by a health care professional; leading to hospitalization or increased level of care; or prompting evaluation.

Type 3a: overt bleeding plus a hemoglobin drop of 3 to 5 g/dL* (provided the hemoglobin drop is related to bleed); any transfusion with overt bleeding.

Type 3b: overt bleeding plus a hemoglobin drop of 5 g/dL (provided the hemoglobin drop is related to bleed); cardiac tamponade; bleeding requiring surgical intervention for control (excluding dental, nasal, skin, and hemorrhoid); bleeding requiring intravenous vasoactive agents.

Type 3c: intracranial hemorrhage (does not include microbleeds or hemorrhagic transformation, does include intraspinal); subcategories confirmed by autopsy or imaging, or lumbar puncture; intraocular bleed compromising vision.

Type 4: coronary artery bypass grafting-related bleeding; perioperative intracranial bleeding within 48 hours; reoperation after closure of sternotomy for the purpose of controlling bleeding; transfusion of 5 U of whole blood or packed red blood cells within a 48-hour period; chest tube output 2 L within a 24-hour period.

Type 5a: probable fatal bleeding; no autopsy or imaging confirmation but clinically suspicious.

Type 5b: definite fatal bleeding; overt bleeding or autopsy, or imaging confirmation.

© 2023 Hariri E et al. *JAMA Network Open.*

eFigure Legends

eFigure 1.

Title: Love plot for covariate balance in the 1:1 propensity matched group of aspirin and non-aspirin users.

Caption: We performed 1:1 propensity matching for aspirin and non-aspirin users according to demographic and clinical baseline characteristics as well as baseline diameter, and we achieved a balanced covariate balance depicted on the Love plot here, with an absolute standardized mean difference between -0.1 and 0.1.

eFigure 2.

Title: Kaplan-Meier curves of the cumulative incidence of study outcomes in a 1:1 propensity-matched cohort.

Caption: (A) all-cause mortality, composite of aneurysm repair, rupture, or dissection (B) and (C) Major bleeding. In a 1:1 propensity matched group of patients, there was not significant difference in the risk of all-cause mortality or composite of aneurysm repair, rupture, or dissection according to aspirin use.

eTable 1. *ICD-10* Codes for Included Patient Variables

Variable	ICD10 codes
**Hypertension**	I10
**Alcohol use**	F10.9<br>F10.92<br>F10.95<br>F10.96<br>F10.97<br>F10.98<br>F10.99
**Diabetes**	E08<br>E10<br>E11<br>E13<br>(0,1,2,3,4,5,6,8,9)
**Hypertension**	I10<br>I15

© 2023 Hariri E et al. *JAMA Network Open.*

**Coronary artery disease**	I25.1<br>I25.2<br>I25.5<br>I25.6<br>I25.7<br>I25.8<br>I25.9<br>I25.10<br>I25.11<br>I25.4<br>I25.7<br>I25.810<br>I25.82
**Peripheral vascular disease**	I70.92
**Hyperlipidemia**	E78.0<br>E78.01<br>E78.2<br>E78.4<br>E78.5
**Stroke/TIA**	Z86.73<br>I63.0<br>I63.1<br>I63.2<br>I63.3<br>I63.4<br>I63.5<br>I63.6<br>I63.8<br>I63.9<br><br>I65.0<br>I65.1<br>I65.2<br>I65.8<br>I65.9<br><br>I66.0<br>I66.1<br>I66.2<br>I66.3<br>I66.8<br>I66.9
**Smoking** (Tobacco use disorder)	Z72.0

© 2023 Hariri E et al. *JAMA Network Open.*

**Connective tissue diseases**<br>*Marfan syndrome*<br>*Ehlers-Danlos*<br>*Hereditary hemorrhagic telangiectasia (HHT)*<br>*Anomaly of the peripheral vascular system, unspecified site*<br>*Anomalies of other specified sites of peripheral vascular system*<br>*Other congenital anomalies of circulatory system*<br>*Coarctation of aorta*<br>*Unspecified anomaly of circulatory system*<br>*Personal history of other diseases of the musculoskeletal system and connective tissue*<br>*Family history of other musculoskeletal diseases*<br>*Unspecified diffuse connective tissue disease*<br>*Hypermobility syndrome*<br>*Arterial tortuosity syndrome (ATS)*	 Q87. 4<br>Q79.6<br>I78.0<br>Q27.9<br>Z87.39<br>Z82.69<br>M36.8<br>M35.9<br>M35.8<br>M35.7<br>Q27.8<br>Q25.1<br>Q87.82
**Coagulopathy**<br>*Acquired coagulation factor deficiency*<br>*Other and unspecified coagulation defects*<br>*VWD*<br>*Qualitative platelet defects*<br>*Factor deficiencies*<br>*Arterial embolism and thrombosis*	D68.4<br>D68<br>(0,1,2,3,4,5,6,8,9)<br>D69.1<br>D66<br>D67<br>D68.1<br>D68.4
**Chronic liver disease**	K70<br>K76<br>K76.9<br>K74<br>(0,1,2,3,4,5,6)<br>K70.3
**Chronic kidney disease**	I12<br>I13<br>N18<br>(1,2,3,5,9)
**Congestive heart failure**<br>*Congestive heart failure, unspecified*<br>*Left heart failure*<br>*Systolic heart failure*<br>*Diastolic heart failure*<br>*Rheumatic heart failure (congestive)*	I50<br>(1,2,3,4,9)<br>I11.0<br>I11.9<br>I09.91<br>I13.0<br>I13.1<br>I13.2
**Valvular heart disease**<br>*394.0 Mitral stenosis*	I05<br>I06

© 2023 Hariri E et al. *JAMA Network Open.*

394.2 Mitral stenosis with insufficiency<br>*424.0 Mitral valve disorders*<br>*424.1 Aortic valve disorders*<br>*395.0 Rheumatic aortic stenosis*<br>*398.90 Rheumatic heart disease, unspecified*	I08<br>I34<br>I35<br>I36<br>I37

© 2023 Hariri E et al. *JAMA Network Open.*

eTable 2. Univariate and Multivariable-Adjusted Regression Analyses of All-Cause Mortality and Composite of Aneurysm Repair, Dissection, or Rupture, According to Aspirin Use

	**Unadjusted**	**Multivariable adjusted** †
**Group**	**HR (95% CI)** *	**HR (95% CI)**
**All-cause mortality**		
Overall population	0.85 (0.74, 0.98)	0.92 (0.79, 1.07)
Males	0.86 (0.73, 1.01)	0.94 (0.79, 1.12)
Females	0.81 (0.61,1.09)	0.88 (0.65, 1.19)
Smokers	1.06 (0.77, 1.46)	1.12 (0.80, 1.59)
Non-smokers	0.80 (0.69, 0.94)	0.88 (0.75, 1.04)
**Major Bleeding**		
Overall population	0.96 (0.83, 1.10)	0.95 (0.85, 1.18)
Males	1.02 (0.87, 1.21)	0.93 (0.83, 1.17)
Females	0.77 (0.58,1.01)	0.61 (0.45, 1.29)
Smokers	0.98 (0.76, 1.26)	0.85 (0.65, 1.11)
Non-smokers	0.95 (0.81, 1.13)	0.91 (0.76, 1.08)
**Composite Outcome**		
Overall population	1.13 (0.96, 1.32)	1.16 (0.93, 1.45)
Males	1.13 (0.95, 1.33)	1.20 (0.87, 1.38)
Females	1.01 (0.71, 1.42)	1.05 (0.69, 1.49)
Smokers	0.88 (0.68, 1.15)	0.78 (0.61, 1.21)
Non-smokers	1.21 (1.01,1.45)	1.22 (0.98, 1.49)

*Hazard ratios represent relative effect of aspirin versus no aspirin

†Adjusted for age, sex, smoking, comorbidities (hypertension, diabetes, chronic kidney disease, coronary artery disease, congestive heart failure, anemia), medications (aspirin, statins, beta blockers, metformin calcium channel blockers, ACE inhibitors/ARBs, anticoagulants, P2Y12 receptor inhibitors), and baseline diameter

For all-cause mortality, Cox proportional hazards model was used. For the composite outcome, competing risks model was used with mortality as the competing event.

© 2023 Hariri E et al. *JAMA Network Open.*

eTable 3. Univariate and Multivariable Linear Regression Analyses for the Annualized Change Abdominal Aortic Aneurysm Diameter According to Antiplatelet Use

	Mean annualized change in mm/year (SD)	Unadjusted	Multivariable adjusted †	
**Group**		**Coefficient***	**Coefficient**	***P***
**Overall population**	3.2 (3.8)			
Aspirin (n = 2,150)	2.8 (3)	-0.052	-0.041	0.008
No Aspirin (n = 1,285)	3.8 (4.2)			
**Smokers**				
Aspirin (n = 545)	3.4 (5.1)	-0.044	-0.031	0.29
No Aspirin (n = 334)	4.1 (5.6)			
**Non-smokers**				
Aspirin (n = 1,605)	2.6 (3.5)	-0.041	-0.043	0.016
No Aspirin (n = 951)	3.7 (5.2)			
**Males**				
Aspirin (n = 1,667)	2.5 (4)	-0.041	-0.039	0.027
No Aspirin (n = 1,005)	3.8 (5.3)			
**Females**				
Aspirin (n = 483)	2.7 (3.9)	-0.036	-0.033	0.31
No Aspirin (n = 280)	3.6 (4.8)			
**Aortic diameter 3-4 cm**				
Aspirin (n = 1,500)	2.8 (4.0)	-0.041	-0.041	0.02
No Aspirin (n = 806)	3.8 (7.3)			
**Aortic diameter 4-5 cm**				
Aspirin (n = 573)	2.8 (3.5)	-0.051	-0.047	0.045
No Aspirin (n = 379)	3.5 (5.1)			
**Aortic diameter ≥ 5 cm**				
Aspirin (n = 216)	2.5 (2.0)	-0.043	-0.044	0.43
No Aspirin (n = 165)	3.4 (3.8)			

* Linear regression for square root transformed annualized change in aortic aneurysm diameter

†Adjusted for age, sex (except in males and females, smoking (except in smokers and non-smokers), comorbidities (hypertension, diabetes, chronic kidney disease, dialysis, coronary artery disease, congestive heart failure, anemia), medications (aspirin, statins, beta blockers, metformin, calcium channel blockers, ACE inhibitors/ARBs, anticoagulants, P2Y12 receptor inhibitors), and baseline diameter

© 2023 Hariri E et al. *JAMA Network Open.*

eTable 4. Baseline Characteristics of the Excluded Population

	**No aspirin (N = 84)**	**Aspirin (N = 99)**
**Age, mean (SD)**	73.7 (9.4)	73.9 (9.5)
**Female, n (%)**	19 (23.6)	25 (25.2)
**Initial diameter (cm), median (IQR)**	3.7 (3.3, 4.5)	3.6 (3.3, 4.4)
**Comorbidities, n (%)**		
Smoking	20 (23.9)	24 (24.4)
Hypertension	58 (69.6)	72 (73.4)
Diabetes	73 (23.6)	21 (20.6)
Hyperlipidemia	60 (71.6)	76 (76.9)
Coronary artery disease	31 (36.7)	45 (45.6)
Peripheral vascular disease	25 (30.0)	35 (35.7)
Prior stroke	21 (24.6)	25 (25.3)
Congestive heart failure	14 (16.5)	17 (17.4)
Valvular heart disease	12 (13.7)	16 (15.9)
Chronic kidney disease	17 (20.2)	19 (18.8)
Chronic lung disease	6 (4.0)	4 (4.1)

© 2023 Hariri E et al. *JAMA Network Open.*

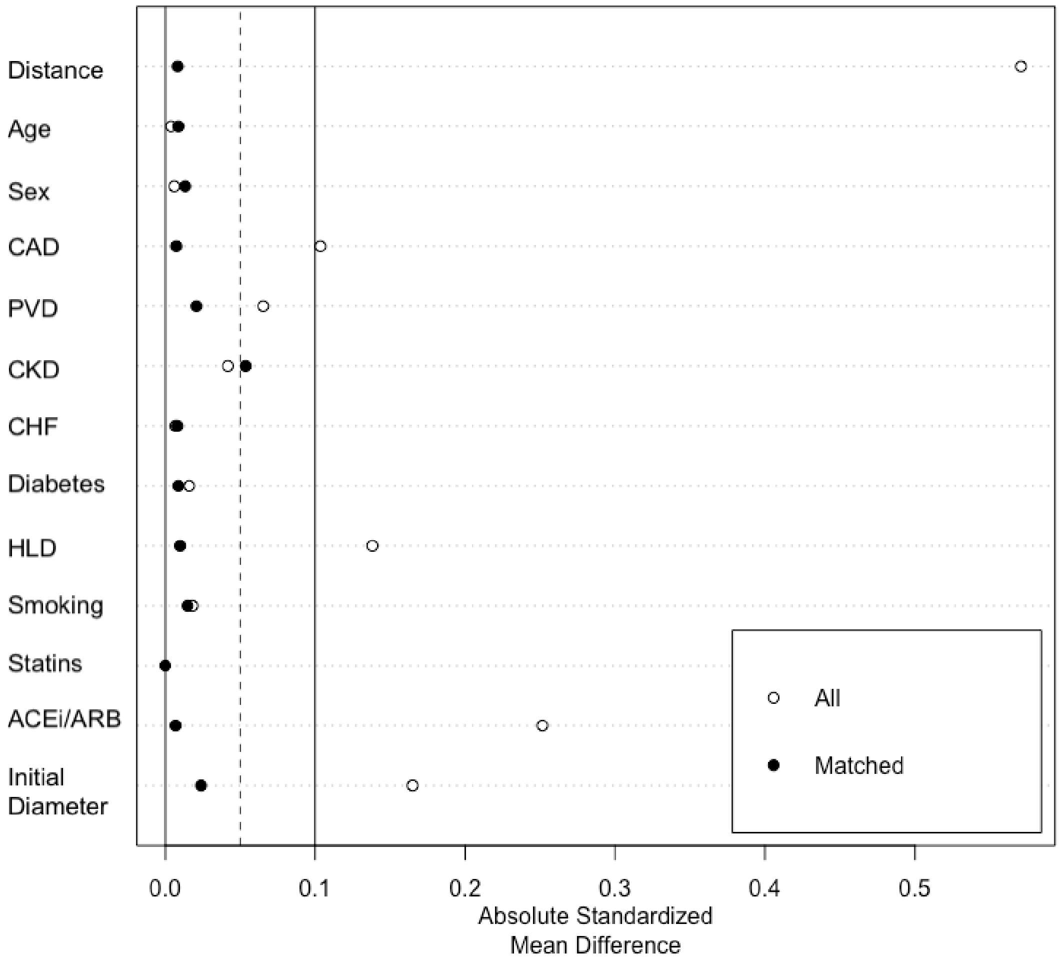

eFigure 1. Love Plot for Covariate Balance in the 1:1 Propensity-Matched Group of Aspirin and Non-Aspirin Users.

We performed 1:1 propensity matching for aspirin and non-aspirin users according to demographic and clinical baseline characteristics as well as baseline diameter, and we achieved a balanced covariate balance depicted on the Love plot here, with an absolute standardized mean difference between -0.1 and 0.1.

© 2023 Hariri E et al. *JAMA Network Open.*

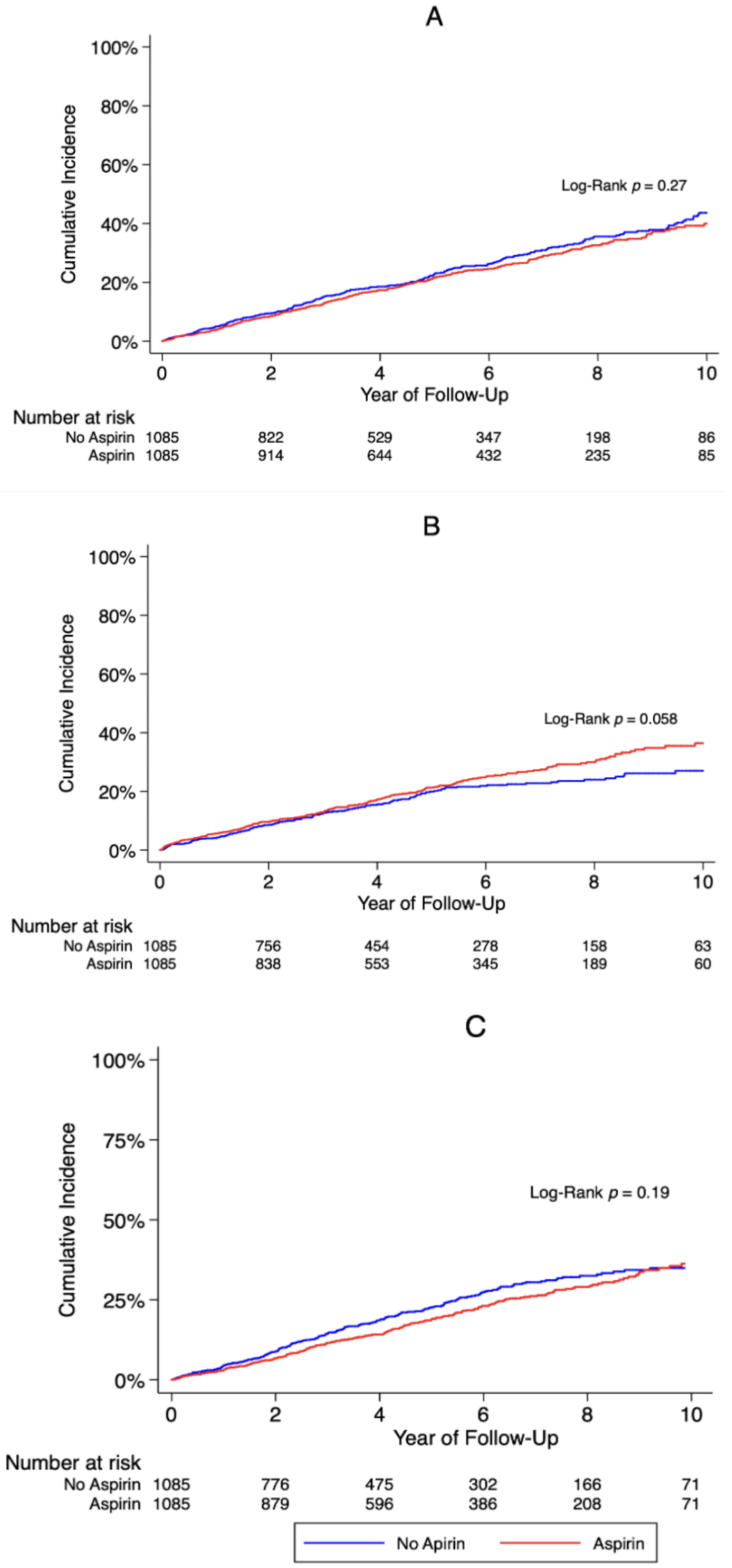
A
100%
80%
60%
40%
20%
0%
Cumulative Incidence
Log-Rank p = 0.27
0
2
4
6
8
10
Year of Follow-Up
Number at risk
No Aspirin 1085 822 529 347 198 86
Aspirin 1085 914 644 432 235 85
B
100%
80%
60%
40%
20%
0%
Cumulative Incidence
Log-Rank p = 0.058
0
2
4
6
8
10
Year of Follow-Up
Number at risk
No Aspirin 1085 756 454 278 158 63
Aspirin 1085 838 553 345 189 60
C
100%
75%
50%
25%
0%
Cumulative Incidence
Log-Rank p = 0.19
0
2
4
6
8
10
Year of Follow-Up
Number at risk
No Aspirin 1085 776 475 302 166 71
Aspirin 1085 879 596 386 208 71
No Apirin
Aspirin

eFigure 2. Kaplan-Meier Curves of the Cumulative Incidence of Study Outcomes in a 1:1 Propensity-Matched Cohort.

(A) all-cause mortality, composite of aneurysm repair, rupture, or dissection (B) and (C) Major bleeding. In a 1:1 propensity matched group of patients, there was not significant difference in the risk of all-cause mortality or composite of aneurysm repair, rupture, or dissection according to aspirin use.

© 2023 Hariri E et al. *JAMA Network Open.*
