# Supplementary material for: Antiplatelet Therapy, Abdominal Aortic Aneurysm Progression, and Clinical Outcomes
Source: JAMA Netw Open. 2023 Dec 12;6(12):e2347296. doi: 10.1001/jamanetworkopen.2023.47296 (PMC10716735; doi:10.1001/jamanetworkopen.2023.47296)
Supplement: Supplement 2. — Data Sharing Statement [file jamanetwopen-e2347296-s002.pdf]

# Data Sharing Statement

Hariri. Antiplatelet Therapy, Abdominal Aortic Aneurysm Progression, and Clinical Outcomes. *JAMA Netw Open*. Published December 12, 2023. doi:10.1001/jamanetworkopen.2023.47296

**Data**
**Data available:** Yes
**Data types:** Deidentified participant data, Other (please specify)
**Additional Information:** Code for study
**How to access data:** Held in a secure and encrypted server by Scott J. Cameron at the Cleveland Clinic.
**When available:** beginning date: 12-01-2023

**Supporting Documents**
**Document types:** None

**Additional Information**
**Who can access the data:** Researchers whose proposed use of the data has been approved via a data use agreement (DUA).
**Types of analyses:** Code, primary deidentified data after an approved MTA.
**Mechanisms of data availability:** With a signed data access agreement signed by giving and receiving institution
